# Inclusion of calcium propionate in late gestation protein supplements increases subsequent offspring marbling scores in range beef cows

**DOI:** 10.1093/tas/txaf099

**Published:** 2025-07-22

**Authors:** Robert L Ziegler, Jacki A Musgrave, Kacie L McCarthy, J Travis Mulliniks

**Affiliations:** West Central Research and Extension Center, University of Nebraska, North Platte, NE 69101, USA; West Central Research and Extension Center, University of Nebraska, North Platte, NE 69101, USA; Department of Animal Science, University of Nebraska-Lincoln, Lincoln, NE 68583, USA; Department of Animal and Rangeland Sciences, Oregon State University, Corvallis, OR 97331, USA

**Keywords:** fetal programming, glucogenic precursors, protein supplementation, range cows, reproduction

## Abstract

A 3-yr study evaluated the effect of late gestation supplementation strategy on cow-calf performance, subsequent steer feedlot performance, and carcass characteristics. Mature March-calving crossbred cows (n = 357) were stratified by body weight (BW) and body condition score (BCS) and assigned to one of 4 treatments: 1) no supplementation (**NoSupp**), 2) 0.91 kg/d of a 30% CP distillers-based supplement (DBS) (**Supp**), 3) 0.91 kg/d of a 30% CP DBS with 160 mg/cow/d of monensin (**RUM**; Rumensin 90, Elanco Animal Health), and 4) 0.91 kg/d of a 30% CP DBS with 40 g/cow/d propionate salt (**CaProp**, NutroCal 100, Kemin Industries). Cows were individually supplemented daily using a Super SmartFeed (C-Lock Inc., Rapid City, SD) from November to February. After weaning, steers (n = 181) were transported to the West Central Research and Extension Center and placed in a GrowSafe feeding system for finishing. Steers were slaughtered at a commercial facility (Tyson Fresh Meats, Lexington, NE) after fed to a common endpoint each year. Cow BW was not different (*P* = 0.87) at the initiation of the study in November. However, NoSupp cows were lighter (*P* < 0.01) at pre-calving and pre-breeding compared to supplemented cows. In contrast, NoSupp cows lost less (*P* < 0.01) BW from pre-calving to pre-breeding than supplemented cows and gained more (*P* = 0.01) BW from pre-breeding to weaning. Treatment tended to influence overall pregnancy rates (*P* = 0.09). Supp and CaProp dams had increased pregnancy rates over NoSupp and RUM (*P* ≤ 0.05). Offspring from NoSupp dams had lighter (*P* < 0.01) BW at birth, pre-breeding, and weaning than their counterparts from supplemented dams. In addition, steer feedlot entry, final BW, and hot carcass weight were greater (*P* < 0.01) when dams were supplemented compared to NoSupp. Steer feedlot average daily gain and gain:feed ratio was not influenced (*P* ≥ 0.31) by dam supplementation strategies; however, dry matter intake tended (*P* = 0.09) to be influenced by treatment. Percentage of carcasses grading Choice or greater was not influenced (*P* = 0.66) treatment; however, steers from dams fed CaProp had increased (*P* = 0.04) marbling scores. Protein supplementation during late gestation is an effective strategy to increase cow BW, maintain cow BCS, and increase progeny BW. Providing dams with propionate salts during late gestation positively improved marbling score of the subsequent steer carcass characteristics.

## Teaser

Supplementing beef cows with protein during late gestation while grazing dormant winter range is an effective strategy to improve cow performance, calf body weight, and improve steer calf carcass characteristics. In addition, inclusion of calcium propionate in protein supplements during late gestation increases cow reproductive performance and their offsprings marbling score.

## INTRODUCTION

During late gestation, range beef cows grazing dormant, low-quality forage may experience reduced nutrient intake alongside increased nutrient demands, leading to a negative energy balance. With the decline in forage quality for spring-calving cows in late gestation, glucose metabolism can be altered with grazing beef cows becoming less sensitive to insulin (Waterman et al., 2007). Reduced sensitivity to insulin in muscle and adipose would allow for increased glucose and amino acid delivery to tissues that are insulin independent for glucose uptake (Bell, 1995). Diminished glucose and amino acids uptake by insulin-sensitive tissues can result in an increased mobilization of fatty acids (Boden, 1998; Tardif et al., 2001). This cascade leads to mobilized fatty acids, which may ultimately depress animal energetic efficiency and productivity of the gestating range cow. While peripheral tissues such as skeletal muscle and adipose tissue rely on insulin for glucose uptake, the placenta utilizes predominantly insulin-independent glucose transporters (Ehrhardt and Bell, 1997). In addition, gravid uterine uptake of glucose, amino acids, and O_2_ increases approximately 3- to 5-fold during late gestation to meet the exponentially increasing demands of the developing fetus and uteroplacenta (Reynolds et al., 1986). Maternal circulating glucose decreases in nutrient restricted females between days 202 and 223 of gestation (Redifer et al., 2023).

Protein is often the most limiting nutrient for range beef cows grazing low-quality native forages where previous research has reported protein supplementation during late gestation results in increased cow BW and BCS before calving (Stalker et al., 2006; Mulliniks et al., 2012). Furthermore, cows consuming low-quality range results in a disproportionate ratio of acetate and propionate (A:P) ratio (Cronjé et al., 1991). This disproportionate A:P ratio negatively impacts energy metabolism, decreases insulin sensitivity, and cow-calf performance in range production systems (Waterman et al., 2006; Mulliniks et al., 2011). However, increasing glucogenic potential of supplements has been shown to increase post-ruminal supply of glucose, increase energy metabolism, and increase insulin sensitivity (Waterman et al., 2006; Mulliniks et al., 2011). Therefore, our hypothesis that providing late gestation protein supplementation with increased glucogenic potential would improve cow performance and subsequent steer feedlot performance. Our objective was to determine the effect of late gestation supplementation strategy on cow-calf performance, subsequent steer feedlot performance, and carcass characteristics.

## MATERIALS AND METHODS

All animal handling and experimental procedures were conducted to the guidelines of the Institutional Animal Care and Use Committee of the University of Nebraska (IACUC approval number 1474).

This study was conducted over a 3-yr period (2020 to 2023) utilizing mature range beef cows from the March-calving herd at the University of Nebraska Gudmundsen Sandhills Laboratory (GSL) located near Whitman, NE. Cows (n = 357) were Red Angus/Simmental crossbred cows and were stratified by cow body weight (BW) and body condition score (BCS) and assigned randomly to a late gestation supplementation treatment. Each year, cows were randomly reassigned to supplementation treatment. Supplementation was initiated in December each year and terminated approximately 2 wk before the start of the calving season in February. During the supplemental period, all cows grazed dormant upland native range in one group. Cows were individually supplemented daily by a Super SmartFeed (C-Lock Inc., Rapid City, SD) electronic pasture feeding system. Supplementation treatments were: 1) no supplementation as the negative control (**NoSupp**), 2) 0.91 kg per day of a 30% CP distillers-based supplement (**Supp**), 3) 0.91 kg per day of a 30% CP distillers-based supplement with the inclusion of 160 mg/cow daily of monensin (**RUM**), 4) 0.91 kg per day of a 30% CP distillers-based supplement with the inclusion of 40 g/cow daily of propionate salt (**CaProp**). Cows that failed to visit the Super SmartFeed electronic feeder were included in the negative control group (NoSupp). Supplemental treatments of RUM and CaProp were designed to provide additional glucogenic precursors that may increase nutrient utilization and efficiency impacting both the performance of the cows and developing fetus.

Warm-season grasses dominate upland range pastures at GSL. The primary plants on range pastures include little bluestem (*Andropogon scoparius* [Michx.] Nash), prairie sandreed (*Calamovilfa longifolia* [Hook.] Scribn.), sand bluestem (*Andropogon hallii* Hack.), switchgrass (*Panicum virgatum* L.), sand lovegrass (*Eragrostis trichodes* [Nutt.] Wood), and blue grama (*Bouteloua gracilis* [H.B.K.] ex Griffiths). Long-term average annual precipitation at GSL is 54.09 cm with an SD of 16.60 cm. Upland, native range pastures at GSL were stocked at 0.6 animal unit months (**AUM**).

Cow BW and BCS (1 = emaciated, 9 = obese; Wagner et al., 1988) by palpation were measured and recorded at weaning (November), pre-calving (February), pre-breeding (May) and at pregnancy check/weaning the following year. Two trained technicians were utilized for determination of BCS. Fertile bulls (1 to 17 bull to cow ratio) were introduced for natural service and removed on d 45 of the breeding season. Cow pregnancy diagnosis was detected via transrectal ultrasonography and rectal palpation at weaning each year.

Steer (n = 181) and heifer (n = 176) calves were weighed at birth, pre-breeding in May, and at weaning. At calving, tissue samples were taken to determine parentage of each calf for sire identification by using a commercially available panel of 96 single nucleotide polymorphisms (SNP). Heifers were retained on the ranch after weaning for replacements. Steers were held in a drylot on ad libitum hay for 2 wk postweaning and then shipped to West Central Research and Extension Center (WCREC; North Platte, NE) and entered the feedlot. Steers were placed in a GrowSafe feeding system approximately 4 wk after arrival at WCREC. Following a 10-d acclimation period in the GrowSafe, steers were weighed 2 consecutive days, and the average was the initial feedlot entry BW used in calculating feedlot performance. All steers experienced a 21-d transition period to a common finishing diet of 48% dry rolled corn, 40% corn gluten feed, 7% prairie hay, and 5% supplement. All steers were implanted with 14 mg estradiol benzonate and 100 mg trenbolone acetate (Synovex Choice, Zoetis) at feedlot entry. Approximately 60 to 100 d prior to slaughter, calves were implanted with 28 mg estradiol benzoate and 200 mg trenbolone acetate (Synovex Plus, Zoetis) and weighed. Each year, steers were slaughtered at a commercial facility (Tyson Fresh Meats, Lexington, NE) when estimated visually to have 1.27 cm fat thickness as an entire group over the 12^th^ rib. Carcass data were collected 24 hour post-slaughter and final BW was calculated from hot carcass weight (HCW) based on an average dressing percentage of 63%. Carcass data included HCW, quality grade, marbling score, yield grade, backfat, and longissimus muscle (LM) area.

Data were analyzed as a randomized design using the MIXED procedure (SAS Inst. Inc., Cary, NC, USA). Cow served as experimental unit with supplemental treatment, year, cow age, calf sex, and sire of offspring set as fixed effects. In addition, the initial model included the interactions of treatment × cow age, treatment × year, treatment × year × cow age, and cow age × year. All binomial data were analyzed using PROC GLIMMIX. The model included fixed effects of treatment, year, and cow age. Least squares means and SE of the mean for binomial data were obtained using ILINK function. Significance level was set at *P* ≤ 0.05. The interactions of treatment with year or cow were not found to be significant for any variable and only the effects of supplemental treatment will be discussed.

## RESULTS

### Supplementation Strategy on Cow Performance

Initial cow BW (*P* ≥ 0.87 Table 1) at the start of the trial in November was not different among treatments. Pre-calving cow BW in February was greater (*P* < 0.01) for protein-supplemented cows (Supp, CaProp, and RUM) compared to NoSupp cows with no differences (*P* *>* 0.49) among protein supplemented cows. At pre-breeding in May, NoSupp cows had the lightest (*P* < 0.01) BW with no difference (*P* ≥ 0.36) among cows that received protein supplementation prepartum. At weaning in November, cow BW was not different (*P* = 0.93) across all late gestation treatment groups.

**Table 1.** Effect of late gestation supplementation on cow body weight, body condition score, and reproductive performance.

	Treatment^1^					
Measurement	NoSupp	Supp	CaProp	RUM	SEM	*P*-value
Cows, n	105	86	80	86		
Cow body weight, kg						
Initial^2^	503	505	504	501	7.3	0.87
Pre-calving^3^	490^b^	535^a^	535^a^	529^a^	6.8	< 0.01
Pre-breeding^4^	473^b^	499^a^	491^a^	491^a^	6.8	< 0.01
Weaning^5^	513	519	513	512	6.8	0.93
Body weight change, kg						
Initial to Pre-calving	−12.7^b^	29.1^a^	30.9^a^	27.3^a^	5.45	< 0.001
Pre-calving to Pre-breeding	−17.7^a^	−35.5^b^	−43.6^b^	−37.3^b^	5.91	< 0.01
Pre-breeding to Weaning	40.0^a^	20.0^b^	22.3^b^	20.5^b^	5.00	0.01
Cow BCS						
Initial	5.3	5.4	5.4	5.4	0.05	0.18
Pre-calving	4.8^b^	5.3^a^	5.4^a^	5.3^a^	0.06	< 0.01
Pre-breeding	5.0^b^	5.3^a^	5.3^a^	5.3^a^	0.05	< 0.01
Weaning	5.3	5.4	5.4	5.5	0.33	0.46
Pregnancy rate, %	90^b^	96^a^	95^a^	91^b^	--	0.09

^1^Treatment = No supplementation (NoSupp), 0.91 kg/d of a 30% crude protein distillers-based supplement (Supp), 0.91 kg/d of a 30% crude protein distillers-based supplement with 160 mg/cow/d of monensin (RUM; Rumensin 90, Elanco Animal Health, 0.91 kg/d of a 30% crude protein distillers-based supplement with 40 g/cow/d propionate salt (CaProp; NutroCal 100, Kemin Industries).

^2^Initial BW = BW recorded at weaning in November prior to the initiation of late gestation supplementation treatments.

^3^Pre-calving = BW recorded in February.

^4^Pre-breeding = BW recorded in May.

^5^Weaning = BW recorded in November.

^ab^For each treatment interaction, means in rows with different superscripts differ (*P* ≤ 0.05).

Cow BW change from the initiation of supplementation to pre-calving was influenced (*P* < 0.001) by late gestation treatment. Protein-supplemented cows (Supp, CaProp, and RUM) increased (*P* ≤ 0.001) BW from trial initiation to pre-calving whereas NoSupp cows lost BW during the same time period with no differences (*P* ≥ 0.79) in BW change among protein-supplemented cows. All cows lost weight between pre-calving and pre-breeding. However, NoSupp cows lost less (*P* ≤ 0.01) BW from pre-calving to pre-breeding than protein-supplemented cows with no difference (*P* ≥ 0.38) in BW loss among protein-supplemented cows. From pre-breeding to weaning, NoSupp cows gained more BW (*P* = 0.01) than their supplemented counterparts with no difference (*P* ≥ 0.88) in the amount of BW gain between protein-supplement treatment groups.

At the initiation of the trial, cow BCS was similar (*P* = 0.18; Table 1) among the late gestation treatment groups. However, at pre-calving and pre-breeding, cow BCS was influenced (*P* < 0.01) by late gestation supplementation. At both time points, cow BCS was not different (*P* ≥ 0.12) among cows that received protein supplementation; however, NoSupp cows were thinner (*P* ≤ 0.001) than their protein-supplemented counterparts at pre-calving and pre-breeding. By weaning in Nov, cow BCS was not different (*P* = 0.46) among late gestation treatments.

Late gestation supplementation strategy tended (*P* = 0.09; Table 1) to influence overall pregnancy rates with pregnancy rates being greater (*P* ≤ 0.05) for Supp and CaProp-supplemented cows than NoSupp and RUM-supplemented cows.

### Dam Supplementation Strategy on Pre-Weaning Calf Growth

Calf BW at birth was influenced (*P* < 0.01; Table 2) by dam’s late gestation supplementation treatment. Calves born from cows in the NoSupp treatment group had the lightest (*P* ≤ 0.01) calf BW at birth compared to their protein-supplemented counterparts with no difference (*P* ≥ 0.42) in calf birth BW among protein-supplemented treatment groups. Calf BW at pre-breeding in May and weaning responded similarly to calf BW at birth. Calves from NoSupp dams were the lightest (*P* ≤ 0.01) at pre-breeding in May and weaning compared to calves from protein-supplemented dams with no difference (*P* ≥ 0.30) in BW in calves from protein-supplemented dams.

**Table 2.** Impact of late gestation supplementation on calf body weight.

	Treatment^1^					
Measurement	NoSupp	Supp	CaProp	Rum	SEM	*P*-value
Calf body weight, kg						
Birth	33^b^	35^a^	35^a^	35^a^	0.5	< 0.01
Pre-breeding^2^	72^b^	80^a^	79^a^	79^a^	1.8	< 0.01
Weaning	249^b^	261^a^	262^a^	259^a^	2.7	0.01

^1^Treatment = dams were offered one of four treatments, No supplementation (NoSupp), 0.91 kg/d of a 30% crude protein distillers-based supplement (Supp), 0.91 kg/d of a 30% crude protein distillers-based supplement with 160 mg/cow/d of monensin (RUM; Rumensin 90, Elanco Animal Health, 0.91 kg/d of a 30% crude protein distillers-based supplement with 40 g/cow/d propionate salt (CaProp; NutroCal 100, Kemin Industries).

^2^Pre-breeding = May calf body weight.

^ab^For each treatment interaction, means in rows with different superscripts differ (*P* ≤ 0.05).

### Dam Supplementation Strategy on Steer Feedlot Performance and Carcass Characteristics

Steers from protein-supplemented dams had increased (*P* < 0.01; Table 3) feedlot arrival BW and final BW compared to steers from NoSupp dams. In addition, steers from protein-supplemented dams tended (*P* = 0.07) to influence steer BW at 100-d implant with steers from dams who received protein having increased BW as compared to NoSupp. Feedlot average daily gain (ADG) was not influenced (*P* ≥ 0.18) by dam’s prepartum supplementation strategy during the entire feeding period. Dry matter intake (DMI) tended (*P* = 0.09) to be influenced by dam’s prepartum supplementation strategy. Dry matter intake was not different (*P* ≥ 0.12) among steers from protein-supplemented treatment groups. In addition, DMI was not different (*P* ≥ 0.23) between steers from NoSupp, CaProp, and RUM dams; however, steers from NoSupp dams had lower (*P* = 0.01) DMI than steers from Supp dams. Gain to Feed (GF) was not influenced (*P* = 0.49) by dam’s prepartum supplementation strategy.

**Table 3.** Impact of late gestation supplementation on steer progeny feedlot performance and carcass characteristics.

	Treatment^1^					
Measurement	NoSupp	Supp	CaProp	RUM	SEM	*P*-value
Feedlot performance, kg						
Entry	330^b^	355^a^	351^a^	348^a^	7.7	< 0.01
Reimplant	475^b^	497^a^	497^a^	499^a^	10.5	0.07
Finished^2^	565^b^	597^a^	602^a^	599^a^	11.4	< 0.01
Average daily gain, kg/d						
Entry to Reimplant	1.93	1.91	1.96	2.00	0.08	0.62
Reimplant to Finished	1.64	1.82	1.90	1.81	0.11	0.18
Overall average daily gain	1.82	1.92	1.89	1.90	0.06	0.31
Dry matter intake^3^, kg/d	9.83^b^	10.52^a^	10.18^ab^	10.10^ab^	0.277	0.09
Gain:Feed ratio^4^	0.187	0.182	0.183	0.189	0.003	0.49
Carcass Characteristics						
Hot carcass weight, kg	356^b^	376^a^	380^a^	377^a^	7.3	< 0.01
Choice or greater, %	82	83	85	73	--	0.66
Yield Grade	2.73	2.93	2.98	2.82	0.16	0.62
Longissimus muscle area, cm^2^	90.97^c^	93.03^bc^	96.77^a^	94.52^ab^	2.00	0.05
Marbling score	496^b^	498^b^	531^a^	492^b^	10	0.04
Backfat, cm	1.32	1.47	1.50	1.42	0.10	0.41

^1^Treatments = dams were offered one of four treatments, No supplementation (NoSupp), 0.91 kg/d of a 30% crude protein distillers-based supplement (Supp), 0.91 kg/d of a 30% crude protein distillers-based supplement with 160 mg/cow/d of monensin (RUM; Rumensin 90, Elanco Animal Health, 0.91 kg/d of a 30% crude protein distillers-based supplement with 40 g/cow/d propionate salt (CaProp; NutroCal 100, Kemin Industries).

^2^Calculated on a carcass-adjusted basis using a common dressing percentage (63%).

^3^Dry matter intake calculated from entry to finishing.

^4^Gain:Feed ration = overall average daily/dry matter intake.

^abc^For each treatment interaction, means in rows with different superscripts differ (*P* ≤ 0.05).

Similar to previous BW measurements, hot carcass weight (HCW) was influenced (*P* < 0.01; Table 3) by dam’s prepartum supplementation strategy. Steers from protein-supplemented dams had similar (*P* ≥ 0.67) HCW among the 3 different treatments; however, steers from NoSupp dams had lighter (P < 0.01) HCW compared to their counterparts from protein-supplemented dams. Dam’s prepartum supplementation strategy did not influence the percentage of carcasses grading Choice or greater (*P* = 0.66), yield grade (*P* = 0.62), nor backfat thickness (*P* = 0.41). Longissimus muscle (LM) area was influenced (*P* = 0.05) by previous dam’s prepartum supplementation treatment. Steers from CaProp and RUM dams had similar (*P* = 0.31) LM areas; however, steers from CaProp and RUM dams did have larger (*P* ≤ 0.05) LM areas than NoSupp offspring. Longissimus muscle area for CaProp steers tended (*P* = 0.09) to be larger than Supp steers. In addition, LM area was not different (*P* = 0.47) between steers from Supp and RUM dams. Steer progeny marbling score was influenced (*P* = 0.04) by dam’s prepartum supplementation strategy. Marbling score was increased (*P* ≤ 0.04) in CaProp steers compared to their counterparts with no difference (*P* ≥ 0.80) between NoSupp, Supp, and RUM steers.

## DISCUSSION

### Supplementation Strategy on Cow Performance

Due to increased nutrient requirements and a decrease in nutrient quality of grazable forage during late gestation, cows may have to mobilize energy reserves and go into a negative energy balance to meet the increased nutrient requirement associated with the rapidly developing fetus. In low-quality native range, protein quantities tend to be more limiting to grazing animal performance than energy (Wallace, 1987), which protein supplementation can improve DMI and digestibility of dormant, low-quality forages (McCollum and Horn, 1990) and improve cow BCS and BW gain during late gestation (Mulliniks et al., 2012). In the current study, all protein-supplemented treatment groups gained BW (27 to 30 kg) during late gestation while NoSupp cows lost 12.7 kg during that same period of time. As a result of the late gestation BW change, NoSupp cows lost BCS from initiation of supplementation to calving (5.3 to 4.8; respectively) while all protein-supplemented cows maintained BCS. Similarly, protein supplementation compared to no supplementation during late gestation has been previously shown to increase cow BW while maintaining BCS (Stalker et al., 2006; Mulliniks et al., 2012; Bohnert et al., 2013), which all 3 of these studies illustrate a similar BW loss as the current study of 14 to 28 kg with non-supplemented cows during late gestation.

Late gestation protein supplementation with or without the inclusion of calcium propionate (CaProp) or monensin (RUM) all responded similarly for cow BW, BW change and BCS. Similarly, Linneen et al. (2015) reported monensin inclusion in a protein supplement during late gestation did not influence cow BW change. In addition, Jacques et al. (1987) supplemented gestating beef cows grazing dormant tallgrass prairie with four levels of lasalocid, 0, 100, 200, 300 mg ∙ hd^−1^ daily and reported no differences in cow BW change. Similar to calcium propionate used in the current study, propylene glycol is a precursor for ruminal propionate. Chibisa et al. (2008) reported no impact on cow BW from propylene glycol inclusion in the diet when fed to dairy cows during late gestation and into early lactation. Previous studies feeding calcium propionate with protein during early lactation in range beef cows also reported no positive impact cow BW or BW change (Waterman et al., 2006; Mulliniks et al., 2011).

Late gestation supplementation strategies have been highly variable on the impact to subsequent pregnancy rates. Broadhead et al. (2019) illustrated an increase in pregnancy rates when cows were offered a protein supplement during late gestation compared to no supplement. In contrast, other studies have reported no impact on cow reproductive performance due to late gestation protein supplementation (Stalker et al., 2006; Larson et al., 2009; Mulliniks et al., 2012). In the current study, late gestation supplementation strategy tended to impact subsequent cow pregnancy rate with CaProp and Supp cows having greater pregnancy rates than RUM and NoSupp cows with no difference in pregnancy rate between RUM and No Supp cows. Supplementing fall-calving cows during late gestation with 200 mg/d of monensin or without monensin has been reported to have similar pregnancy rates between the two groups (Vedovatto et al., 2022). In a meta-analysis, Gadberry et al. (2022) reported feeding monensin to cows prior to breeding increased the proportion of cows exhibiting estrus and reduced the days to resumption of first estrus, but offering monensin to mature cows did not influence overall pregnancy rates. Similar results as in the current study have been reported in lactating cows feeding propionate salts or monensin after calving. In young, lactating range cows, King et al. (2023) reported increased pregnancy rates and percentage of cows cycling prior to the start of breeding with propionate salt inclusion into protein supplements compared with monensin. In addition, postpartum protein supplementation with propionate salt inclusion has been reported to decrease days to resumption of estrus and have a positive impact on overall pregnancy rates in young range cows (Mulliniks et al., 2011).

### Dam Supplementation Strategy on Pre-Weaning Calf Growth

In the current study, protein supplementation during late gestation with or without feed additives resulted in increased calf BW at birth, branding, and at weaning compared to non-supplemented cows. In agreement, Bohnert et al., (2013) reported increased calf BW at birth and weaning when dams were supplemented with dried distillers grains with solubles (DDGS) compared to non-supplemented cows during late gestation. Winterholler et al., (2012) implied that added energy from DDGS supplementation may have partitioned nutrients to help support fetal growth resulting in increased calf BW at birth compared to non-supplemented dams. In agreement with the current study, protein supplement with an ionophore did not influence calf BW at birth as compared to protein supplementation without an ionophore (Jacques et al., 1987; Vedovatto et al., 2022).

Protein-supplementation during late gestation resulted in increased subsequent calf BW at weaning compared to offspring from non-supplemented cows. In agreement with the current study, previous research has shown that late gestation protein supplementation can increase calf BW at weaning compared to offspring from non-supplemented dams (Stalker et al., 2006; Broadhead et al., 2019). Vedovatto et al. (2022) attributed an increase in preweaning calf growth performance to the increase in BCS of cows that were supplemented in addition to increased plasma IGF-1 concentrations. The inclusion of feed additives in protein supplements in the current study during late gestation did not result in differences in calf BW at weaning. In agreement, Linneen et al. (2015) reported no differences in calf 205-d adjusted weaning weight when monensin was fed to beef cows in late gestation and early lactation. In addition, post-partum supplementation of protein supplements with either monensin or calcium propionate did not result in differences in calf BW at weaning (King et al., 2023).

### Dam Supplementation Strategy on Steer Feedlot Performance and Carcass Characteristics

Similar to the pre-weaning calf growth, steer feedlot entry BW and live final BW were increased in steers from protein-supplemented dams with no differences among protein-supplemented treatment groups. In contrast, Mulliniks et al. (2012) reported no differences in feedlot entry and final BW in steers from protein supplemented or non-supplemented dams. In a concurrent study to the current study at GSL, Musgrave et al. (2025) reported no differences in steer feedlot entry and final BW from protein-supplemented or variable-supplemented dams. In support of the current study, Larson et al. (2009) reported increased steer feedlot entry and final live BW when late gestating dams were fed protein supplements.

After the finishing phase, steer HCW was increased in steers from protein-supplemented dams compared to non-supplemented dams with no differences among the 3 protein supplement treatment groups. In agreement, Larson et al. (2009) reported a tendency for increased steer HCW from protein-supplemented dams during late gestation. However, the response of late gestation supplementation strategies on offspring HCW has been highly variable. In a review, Moriel et al. (2021) indicated that other factors during gestation may have a larger impact on final offspring performance than maternal nutrition. In contrast to the current study, previous research has shown no impact of late gestation supplementation strategy on subsequent offspring HCW (Mulliniks et al., 2012; Radunz et al., 2012; Bohnert et al. 2013; Maresca et al., 2019; Musgrave et al., 2025).

In a review, Moriel et al. (2021) summarized that the majority of late gestation supplementation studies show minimal to no impact of supplementation strategy/nutrient restriction on offspring LM area after the finishing phase. However, mid- to late gestating cows consuming a low-quality forage-based diet at 6% CP crude protein (DM basis) has been shown to decrease subsequent steer LM area compared to offspring born from cows consuming a 12% CP diet without impacting HCW or backfat thickness (Maresca et al., 2019). In the current study, steer LM area was influenced by the dam’s late gestation strategy. Steers from CaProp and RUM dams had greater LM area than steers from NoSupp dams. In addition, steers from CaProp dams tended to have greater LM area than steers from Supp dams. Steers from RUM and Supp dams had similar LM area and no differences between steers from NoSupp and Supp. These results do illustrate a potential benefit of feeding glucogenic precursors during late gestation on increasing LM area in subsequent offspring. Feeding calcium propionate to gestating ewes has been shown to improve offspring lean tissue texture and increase unsaturated fatty acids in lean muscle (Pérez Sequara et al., 2023).

In the current study, inclusion of calcium propionate in protein supplements during late gestation increased subsequent offspring marbling score with no differences among the other strategies, which includes no supplementation during late gestation. In contrast, Larson et al. (2009) reported an increase in marbling scores of steers from protein-supplemented dams during late gestation compared to non-supplemented. However, previous studies have shown no impact of late gestation supplementation strategy including no supplementation on subsequent offspring marbling score (Stalker et al., 2006; Mulliniks et al., 2012; Bohnert et al., 2013; Maresca et al., 2019; Musgrave et al., 2025). In dairy cows, maternal insulin resistance during late gestation is associated with altered metabolic traits in the subsequent pre-weaned calf with increased plasma insulin concentrations and similar serum glucose concentrations in offspring from insulin resistant cows (Kawashima et al., 2016). Thus, supplementation strategies resulting in decreased insulin resistance during late gestation may impact post-natal growth and development of subsequent offspring. Late gestation dietary energy sources have been shown to alter fetal adipose tissue development and insulin sensitivity resulting in change in offspring marbling score where cows consuming a starch-based diet resulting in increased insulin resistance during late gestation and decreased marbling score of offspring (Radunz et al. 2012). As forage quality declines, maternal tissues become less responsive to insulin resulting in late gestating cows becoming insulin resistant (Waterman et al., 2007), which may exacerbate nutritional imbalance in range production settings. In lactating range cows, increasing glucogenic precursors supply has resulted in increased insulin sensitivity (Waterman et al., 2006) and energy utilization (Mulliniks et al., 2011). In addition, increasing glucose supply stimulates cell proliferation, abundance of glucose transporter GLUT1 and GLUT4, and metabolism by mediating the mTOR signaling pathway in bovine placental trophoblast cells (Shi et al., 2023). Therefore, the increase in dietary glucogenic potential in the form of propionate salts may increase the amount of glucose available to the gravid uterus. During late gestation, increased fetal glucose supply promotes fetal adiposity in lambs ( Stevens et al., 1990). Glucose availability during this period is considered the primary regulator of lipid accumulation in fetal adipocytes in sheep (Mühlhäusler et al., 2003, 2005). Glucose has been shown to enhance lipid accumulation and stimulate the expression of adipogenic genes, including the upregulation of peroxisome proliferator-activated receptor gamma (PPARγ), a key regulator of adipogenesis (Kolodziej et al., 2019). The increase in marbling scores in steers from dams supplemented with CaProp during late gestation could be attributed to an increase in glucose supplied to the fetus, increasing the number of adipocytes and therefore improving marbling scores.

## CONCLUSIONS

Inconsistent results exist regarding the impacts of late gestation protein supplementation in beef cattle; however, results from this study demonstrate that protein supplementation to late gestating range cows grazing dormant winter forage improves cow body weight and enhances calf growth performance through the finishing phase. While the inclusion of monensin did not result in additional benefits beyond protein supplementation alone, the inclusion of calcium propionate shows potential to enhance carcass quality in progeny. These findings underscore the importance of meeting protein requirements during late gestation and highlight increasing glucogenic potential of the diet through calcium propionate as a potential tool for improving carcass traits, particularly marbling and muscle development, in beef production systems.
